# Naturally Occurring Precore/Core Region Mutations of Hepatitis B Virus Genotype C Related to Hepatocellular Carcinoma

**DOI:** 10.1371/journal.pone.0047372

**Published:** 2012-10-10

**Authors:** Dong-Won Kim, Seoung-Ae Lee, Eung-Soo Hwang, Yoon-Hoh Kook, Bum-Joon Kim

**Affiliations:** Department of Microbiology and Immunology, Liver Research Institute and Cancer Research Institute, College of Medicine, Seoul National University, Seoul, Korea; University of Cincinnati College of Medicine, United States of America

## Abstract

Previous studies have proved the presence of several distinct types of mutations in hepatitis B virus (HBV) infections, which are related to the progression of liver disease. However, few reports have detailed the mutation frequencies and mutation patterns in the precore/core (preC/C) region, which are based on the clinical status and HBeAg serostatus. Our aim in this study is to investigate the relationships between the preC/C mutations and clinical severity or HBeAg serostatus from patients chronically infected with HBV genotype C. A total of 70 Korean chronic patients, including 35 with hepatocellular carcinoma (HCC), participated in this study. HBV genotyping and precore/core mutations were analyzed by direct sequencing. All patients were confirmed to have genotype C infections. Mutations in the C region were distributed in a non-random manner. In particular, mutations in the MHC class II restricted region were found to be significantly related to HCC. Six (preC-W28*, C-P5H/L/T, C-E83D, C-I97F/L, C-L100I and C-Q182K/*) and seven types (preC-W28*, preC-G29D, C-D32N/H, C-E43K, C-P50A/H/Y, C-A131G/N/P and C-S181H/P) of mutations in the preC/C region were found to be related to HCC and to affect the HBeAg serostatus, respectively. In conclusion, our data indicated that HBV variants in the C region, particularly in the MHC class II restricted region, may contribute to the progress of HCC in chronic patients infected with genotype C. In addition, we found several distinct preC/C mutations in the Korean chronic cohort, which affect the clinical status of HCC and HBeAg serostatus of patients infected with genotype C.

## Introduction

Despite the availability of an effective vaccine, more than 350 million people worldwide are chronically infected with the hepatitis B virus (HBV), and many people have developed serious liver diseases, such as cirrhosis and hepatocellular carcinoma (HCC) [1]. The Republic of Korea has been recognized as an endemic area for HBV infection. For instance, according to the Korean National Health and Nutrition Survey of 1998, the prevalence of HBsAg was 5.1% in men and 4.1% in women [2].

Based on intergroup divergence of >8% in the complete genome sequence, hepatitis B virus (HBV) strains are classified into eight genomic groups or genotypes, which are designated as A–H [1], [2], [3], [4]. There is increasing evidence that HBV genotypes play a significant role in causing different disease profiles in chronic hepatitis B (CHB) infection [5], [6]. An extraordinary prevalence of genotype C2, which is known to be more virulent than genotype B, has been reported in Korea [7] and is expected to affect the mutation patterns and frequencies of Korean HBV strains. In actuality, relatively high mutation frequencies in the basal core promoter (BCP) [7], [8], [9], [10] and in the major hydrophilic region (MHR) [11] have already been reported in Korean patients.

Over the past decade, increasing attention has been focused on variant HBV strains that contribute to the clinical severity of liver diseases, especially HCC. To date, a number of mutation patterns of HBV, such as the precore mutation at nucleotide 1896 (G→A) or the double mutation in the basal core promoter (BCP) region at nucleotides 1762 (A→T) and 1764 (G→A), have been widely studied as HBV mutations related to clinical severity [12], [13], [14]. The two types of mutations related to clinical severity, the F141L preS2 mutation [15] and W182* leading to premature termination in the HBV surface antigen (HBsAg) [16], were recently noted in Korean chronic patients.

The HBV C protein (HBcAg), the protein shell of the virus core, is 183 residues long, of which 149 residues of the N-terminal are the assembly domain [17], [18]. HBcAg is the principal target for the host immune response, particularly cytotoxic T lymphocyte attack, in which non-synonymous mutations that change immune epitopes may lead to the production of immune escape variants, resulting in the persistence of HBV [19], [20], [21]. Moreover, because a mutation in the C region can lead to simultaneous mutations in HBeAg, a key HBV immune-regulatory protein, the mutation may also profoundly affect the natural course of CHB [22].

Relationships between the frequencies of the preC/C region and the progression of liver disease have been elucidated [23], [24], [25], [26]. However, confirmative determinations of the mutation of a single codon related to HCC or affecting the HBeAg serostatus have rarely, if ever, been reported. Therefore, the aims of the present study were as follows: (1) to elucidate the prevalence of naturally occurring preC/C mutations in Korean HBV patients based on clinical status and HBeAg serostatus, and (2) to determine the characteristic patterns of preC/C mutations related to HCC or affecting the HBeAg serostatus.

## Methods

### Patients

Plasma serum samples were collected from 70 chronic hepatitis B patients who visited the Seoul National University Hospital in 2005. Among these, 35 serum samples were HBeAg-positive and 35 were HBeAg-negative. Clinical diagnoses of the subjects were chronic hepatitis (n = 27), liver cirrhosis (n = 8) and HCC (n = 35). Chronic liver disease definitions were as follows: chronic hepatitis was defined as an elevation or fluctuation of serum ALT over 6 months without any evidence of any other chronic liver disease [27]; liver cirrhosis was diagnosed as having clinically relevant portal hypertension (esophageal varices and/or ascites, splenomegaly with a platelet count of <100,000/mm^3^) [28] and ultrasonographic imaging features suggestive of liver cirrhosis [29]; and HCC was diagnosed either histologically or radiologically based on the presence of a hypervascular liver mass with serum alpha-fetoprotein (AFP) levels exceeding 400 ng/ml [30]. Patients were excluded if they had any of the following: acute hepatitis B, concomitant hepatitis C or D virus infection, any history of antiviral therapy, a history of immunosuppressive therapy, and a history of heavy alcohol drinking. HBsAg, anti-HBs, HBeAg, and anti-HBe were assayed using a commercial enzyme immunoassay kit (Abbott Laboratory, Wiesbaden, Germany). This work was approved by the institutional review board of Seoul National University Hospital (IRB No. C-1110-106-382). The experiment was mainly based on the extracted virion DNA from isolates; hence, the research was done without informed consent and the waiver of informed consent was agreed upon by the IRB. Clinical details of the study's patients are presented in Table S1.

### DNA extraction

200 *μl* of serum from each subject was incubated for 3 hr at 65°C with 600 ml of TES buffer (10 mM Tris-HCl pH 8.0, 5 mM EDTA, 0.5% SDS, and 50 mg of proteinase K). DNA was extracted using phenol/chloroform/isoamylalcohol (50∶49∶1), and DNA pellets were precipitated with isopropyl alcohol. The DNA pellets were solubilized with 20 *μl* TE buffer (10 mM Tris-HCl, 1 mM EDTA, pH 8.0), and 2 *μl* of the purified DNA was used as a PCR template. HBV DNA was determined quantitatively using a Hybrid capture HBV DNA assay kit (Digene, Gaithersburg, MD, USA). The lower limit of detection of the hybrid capture HBV DNA assay was 1.6 pg/ml.

### HBV DNA amplification and sequencing

To analyze the mutation patterns and their frequencies of deletions and insertions in the entire preC/C region, a nested PCR protocol was used. First-round PCR was performed using the sense primer CoreF1 (5′-AAC GAC CGA CCT TGA GGC ATA CTT-3′) and the antisense primer CoreR1 (5′-ATT TGG TAA GGT TAG GAT AGA A-3′) to yield a 1017 bp amplicon between 1682 nt to 2698 nt of the HBV genome. Second-round PCR was performed using the sense primer CoreF2 (5′-GAG TTG GGG GAG GAG ATT AGG TTA-3′) and the antisense primer CoreR2 (5′-CAC TCA GGA TTA AAG ACA G-3′) to yield an 822 bp amplicon between 1734 nt to 2555 nt of the HBV genome. The PCR was initiated in a 50 *μl* PCR mixture containing 1.5 mM MgCl_2_, 200 μM dNTP, and 2.6 U of Expand High Fidelity Taq polymerase. For both rounds, the protocol was to heat to 95°C, have the initial duration last for 10 min with 30 cycles at 95°C (45 sec), 52°C (45 sec) and 72°C (90 sec). A final extension step was then performed at 72°C for 5 min. We used 5 *μl* of the product from the first-round PCR, and the protocol of the second-round PCR was identical to that of the first. The PCR products obtained were analyzed by electrophoresis on 2.5% agarose gels, stained with ethidium bromide, and visualized on a UV transilluminator.

The purified PCR products were directly determined using both primers of the secondary PCR, CoreF2 and CoreR2, and with a dideoxy method that uses a BigDye Terminator Cycle Sequencing Ready reaction, V. 2, and a fluorescent 373A DNA sequencer (Applied Biosystems, Foster City, CA, USA). If two peaks were present at any position within the chromatograph, the dominant peak was utilized.

### HBV genotyping

For genotyping, a phylogenetic analysis based on entire sequences of the entire preC/C region (639 bp) was performed on all 70 HBV strains. The nucleotide sequences of the 70HBV strains were compared with those of 17 reference strains, representing each of the genotypes of A–D including 14 genotype C strains obtained from GenBank [Accession numbers AB031262 (C), AB074755 (C), AB100695 (B), AF068756 (C), AF223957 (C), AF286594 (C), AY123041 (C), AY247030 (C), AY247032 (C), D12980 (C), D16667 (C), D23680 (C), D23682 (C), GQ377616 (C), X01587 (C), X70185 (A) and X72702 (D)]. A mutation was defined through comparisons with the consensus sequence of HBV strains in our cohort and 17 reference strains. Phylogenetic trees were inferred by means of neighbor-joining with MEGA version 4.0.2 [31].

### Statistical analyses

Results were expressed as percentages, means ± SD, or as medians (range). The differences between categorical variables were analyzed using Fisher's exact test or a Chi-square test. For continuous variables, the Student's *t*-test was used when the data showed a normal distribution, or the Mann-Whitney *U* test was used when the data was not normally distributed. The level of significance of each test was adjusted for multiple tests via Bonferroni correction. A *p*-value of <0.05 (two-tailed) was considered to be statistically significant.

## Results

### Distribution of genotypes

The phylogenetic analysis based on all of the 639-bp sequences of the preC/C region indicated that all 70 HBV strains from Korean patients belonged to genotype C, irrespective of their clinical status or HBeAg serostatus (Fig. S1).

### Distribution of HBV preC/C region mutations

**Figure 1:**
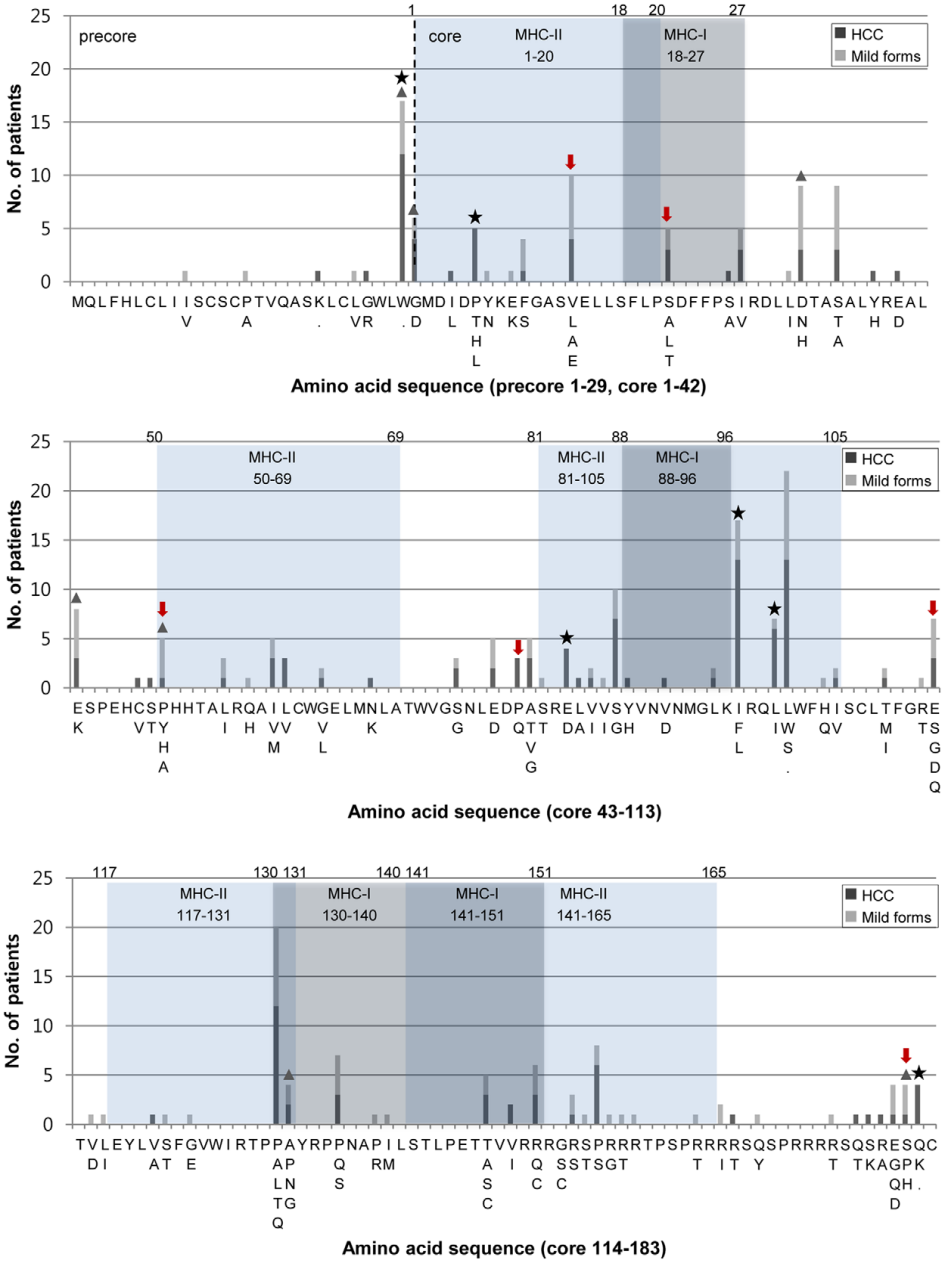
Distribution and frequencies of the amino acid mutations in the preC/C region based on the severity of the liver disease. Black and grey bars represent mutations related to HCC and mild forms of liver disease, respectively. The dark-shaded regions are the MHC class I-restricted (core aa 18–27, 88–96, 130–140, 141–151) and the light-shaded regions are MHC class II-restricted T-cell epitopes of HBcAg (core aa 1–20, 50–69, 81–105, 117–131, 141–165). Asterisks and triangles indicate specific mutations related to HCC and affecting the HBeAg serostatus, respectively, in the present study. The red arrow indicates the six mutations, which were previously reported to be negatively related to HCC [36].

Mutations were observed in a total of 82 out of 212 codons (29 codons in preC and 183 codons in C) from 60 patients out of 70 (85.7%) (Fig. 1, Table 1). Generally, patients with mutations in the preC/C region (60 patients) were significantly older than those without mutations (10 patients) (51.9 vs. 36.9, p<0.001). Other clinical factors that led to significant differences were not found between the two groups, patients with wild type and mutations in the preC/C region (Table 1). No deletions, apart from substitutions, were found in the preC/C region from the 70 patients (data not shown).

**Table 1:** Comparison of the clinical features between patients with the wild type and mutations in the preC/C region.

Clinical factors	Wild type (n = 10)	Mutation (n = 60)^a^	*P*-value
Age in years, mean ± SD	36.9±16.1	51.9±12.7	0.001
Male (%)	7 (70)	46 (76.7)	N.S^b^
HBeAg-positive (%)	6 (60)	29 (48.3)	N.S
Liver disease (no.) CH:LC:HCC^C^	8∶1∶1	19∶7∶34	-
ALT status (%)^d^	6 (60)	47 (81)	N.S
HBV-DNA (pg/ml) median (range)	6.38E+06 (0–5.50E+07)	1.89E+06 (0–8.24E+07)	N.S

a A mutation was defined through comparisons with the consensus sequence of HBV strains in our cohort and 17 reference strains.

b N.S: Not significant.

c CH: Chronic hepatitis, LC: Liver cirrhosis, HCC: Hepatocellular carcinoma.

d The number of patients whose ALT levels were greater than the upper limits of normal ALT levels for men (30 IU/L) and women (19 IU/L) [46].

Generally, a non-random distribution between the mutations of immuno-active region and immuno-inactive regions was shown. The mutation rates in the immuno-active regions (MHC class I + II) were significantly higher than those of the immuno-inactive region (2.2% vs. 1.7%, p = 0.016). The mutation rates in the MHC class II restricted region (designated as M2RR), but not in the MHC class I restricted region (designated M1RR), were significantly higher than those in the immuno-inactive region (2.3% vs. 1.7%, p = 0.009). This contrast was more pronounced considering the mutation rates (4.1%) in “hot spots”, aa residue 81–105 region in M2RR (4.1% vs. 1.7%, p<0.001) (Table 2).

**Table 2:** Comparison of mutation rates between immuno-active and inactive regions of the preC/C region.

Region (n = 14,840)	No. of mutations/No. of codons	Mutation rate (%)	*P*-value^a^
Immuno-inactive regions	107/ 6,370	1.7	-
Immuno-active regions	190/8,470	2.2	0.016
- MHC class I epitope	61/2,870	2.1	N.S^b^
- MHC class II epitope	170/7,350	2.3	0.009^c^
- 81-105 region in MHC class II	72/1,750	4.1	<0.001^c^

a P-values were determined by a comparison with the mutation rate of immuno-inactive regions.

b N.S: Not significant.

c Statistically significant after a Bonferroni post hoc analysis (p<0.05).

Immuno-active regions include MHC class I-restricted epitopes, MHC class II-restricted epitopes, and the 81–105 region of MHC class II.

As expected, mutation in the 28^th^ codon (tryptophan to stop, designated preC-W28*), previously known as the “mutational hot spot” (1896 preC mutation) leading to the inhibition of HBeAg production and related to the progression of liver diseases [32], was found most frequently in the preC region (17 patients, 24.3%). In the C region, mutation in the 101^st^ codon (leucine to tryptophan or serine, designated C-L101W/S) was found the most frequently (22 patients, 31.4%) (Fig. 1).

### Mutation rates between patients with HCC and the comparison group (LC+CH)

The mutation rates of the entire preC/C region in HCC patients (2.2%) tended to be higher than in the comparison group, patients with liver cirrhosis (LC) and those with chronic hepatitis (CH) (1.8%) (p = 0.061). Mutation rates in immuno-active regions, but not in immune-inactive regions were significantly higher in HCC patients than in the comparison group [HCC (2.6%) vs. the comparison group (1.9%), p = 0.033]. Of these, the mutation rates in M2RR (2.7% vs. 1.9%, p = 0.024), but not in M1RR (2.4% vs. 1.8%, p = 0.3), were significantly higher in HCC patients than in comparison group. Furthermore, their difference in the aa residue 81–105 region was also more pronounced (5.6% vs. 2.6%, p = 0.002) (Table 3).

**Table 3:** Comparison of mutation rates of the preC/C region between patients with HCC and the comparison group (LC+CH) in terms of the immuno-active and inactive regions.

Classification	HCC (%)/No. of codons	LC+CH (%)/No. of codons	*P*-value
Immuno-inactive regions	55 (1.7)/3,185	52 (1.6)/3,185	N.S^a^
Immuno-active regions	110 (2.6)/4,235	80 (1.9)/4,235	0.033
- MHC class I epitope	35 (2.4)/1,435	26 (1.8)/1,435	N.S
- MHC class II epitope	100 (2.7)/3,675	70 (1.9)/3,675	0.024
- 81–105 region in MHC class II	49 (5.6)/875	23 (2.6)/875	0.002^b^
Total	165 (2.2)/7,420	132 (1.8)/7,420	0.061

a N.S: Not significant.

b Statistically significant after a Bonferroni post hoc analysis (p<0.05).

### Mutation frequency between patients with two different HBeAg serostatus

Overall, the mutation frequencies in the entire preC/C region in the HBeAg-negative groups (2.5%) were significantly higher than in the HBeAg-positive groups (1.5%) (p<0.001). However, some differences according to respective regions within HBcAg were found. The differences in the mutation rates in M1RR between the two groups (2.6% vs. 1.7%, p = 0.094) did not reach a statistically significant level, However, the mutation rates of M2RR (3.0% vs. 1.7%, p<0.001) were significantly higher in HBeAg-negative patients as compared to HBeAg-positive patients (Table 4).

**Table 4:** Comparison of mutation rates the preC/C region between patients with two different HBeAg serostatus in terms of the immuno-active and inactive regions.

Mutation rate	HBe− (%)/No. of codons	HBe+ (%)/No. of codons	*P*-value
Immuno-inactive regions	67 (2.1)/3,185	40 (1.3)/3,185	0.011
Immuno-active regions	117 (2.8)/4,235	73 (1.7)/4,235	0.002^a^
- MHC class I epitope	37 (2.6)/1,435	24 (1.7)/1,435	0.094
- MHC class II epitope	109 (3.0)/3,675	61 (1.7)/3,675	<0.001^a^
- 81–105 region in MHC class II	44 (5.0)/875	28 (3.2)/875	0.07
Total	184 (2.5)/7,420	113 (1.5)/7,420	<0.001^a^

a Statistically significant after a Bonferroni post hoc analysis (p<0.05).

### Identification of mutation patterns in the preC/C region related to HCC

**Figure 2:**
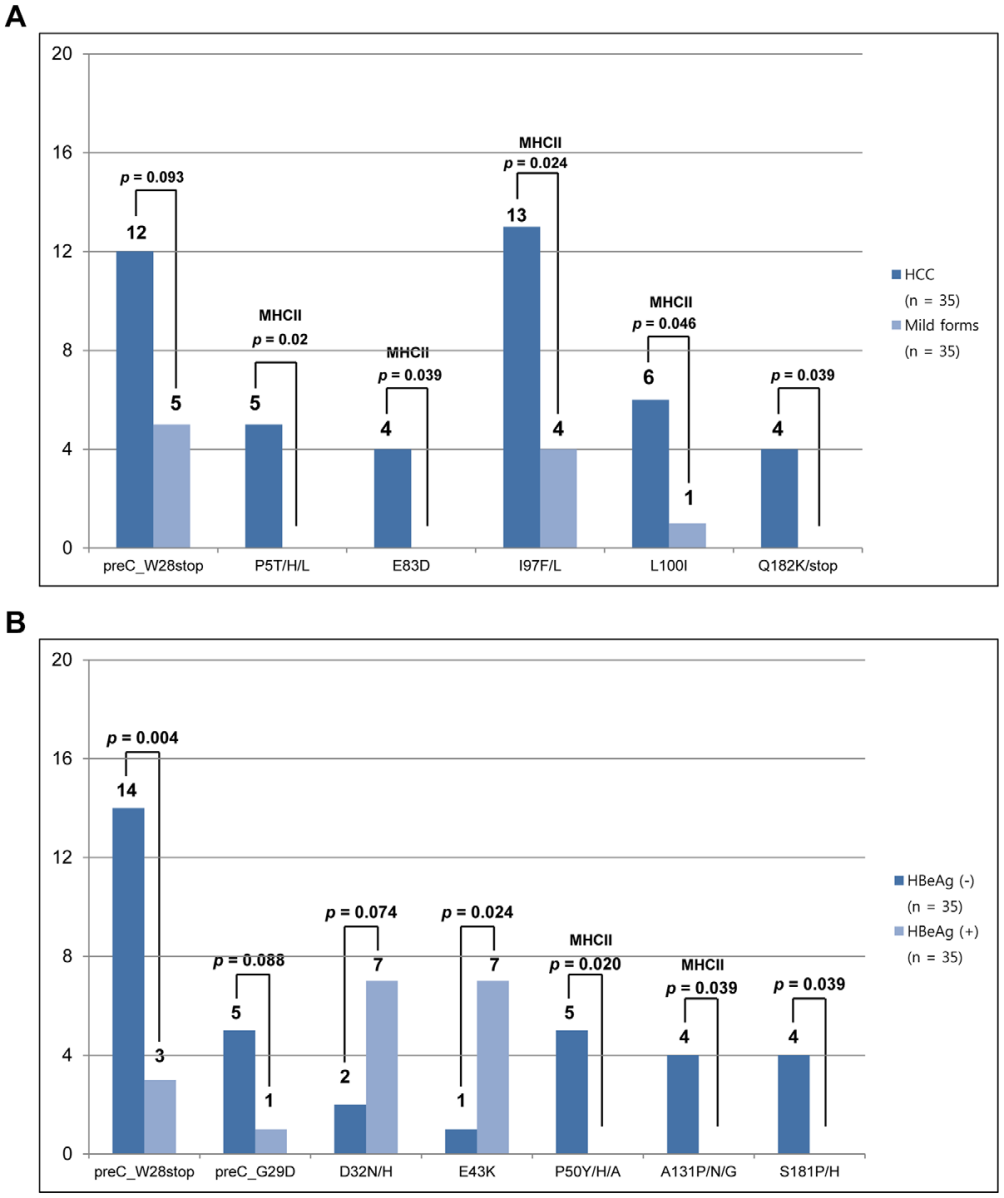
Comparison of frequencies of preC/C mutations according to the severity of liver disease (A) and the HBeAg serostatus (B). The types of mutations within the region of MHC I and II-restricted T-cell epitopes are marked.

Five mutations in the C region (C-P5H/L/T, C-E83D, C-I97F/L, C-L100I and C-Q182K/*) and one in preC (preC-W28*) were found to be related to HCC patients compared to patients at other stages of the disease, such as LC and CH, respectively. Generally, five mutations in the C region were found at a significantly higher level in HCC patients than in patients at other stages of the disease. However, the predominance of preC-W28* in HCC patients did not reach a statistically relevant level (p = 0.093) (Fig. 2a). Notably, the following 4 of 5 HCC-related C mutations, C-P5H/L/T, C-E83D, C-I97F/L and C-L100I, were located at M2RR (one at aa 1–20 and three at aa 81–105, Fig. 1). Among 6 HCC-related preC/C mutations, C-I97F/L, previously known to lead to defects in the HBcAg assembly [33], [34], was found the most frequently in HCC patients (17 HCC patients). A comparison of the clinical data between patients with or without C-I97F/L showed that this mutation was related to old age (57.4 vs. 47.3, p = 0.01) and a high incidence of HCC [wild type (41.5%) vs. I97F/L (76.5%), p = 0.024] (Table S2).

### Identification of mutation patterns in the preC/C region affecting the HBeAg serostatus

Five mutations in the C region (C-D32N/H, C-E43K, C-P50A/H/Y, C-A131G/N/P and C-S181H/P) and two in preC (preC-W28* and preC-G29D) were found to affect the HBeAg serostatus (Fig. 2b). Of those, two mutations (C-D32N/H, C-E43K) and five other mutations (preC-W28*, C-G29D, C-P50A/H/Y, C-A131G/N/P and C-S181H/P) were found to be related to the HBeAg-positive and HBeAg-negative serostatus, respectively. Of these, the frequencies of five mutations (preC-W28*, C-E43K, C-P50A/H/Y, C-A131G/N/P and C-S181H/P), but not two others (C-G29D and C-D32N/H), reached a statistically significant level. Interestingly, the two negatively related HBeAg mutations in the C region (C-P50A/H/Y and C-A131G/N/P) were located at M2RR (Fig. 2b).

## Discussion

Our data showed that the majority of patients (60/70 patients, 85.7%) had more than one mutation in the preC/C region (Table 1). The high frequency of preC/C mutations in the Korean cohort may be due to the nature of the genotype C infection, which is more prone to mutations [6] and the extraordinary predominance of perinatal infection in Korean patients over a horizontal transmission [2]. Our previous studies which used cohorts of chronic Korean patients strongly support this hypothesis [9], [15], [35].

Our data link four major factors, old age, the location of the T cell epitope (particularly M2RR), the HBeAg negative serostatus, and the HCC clinical status, to higher mutation rates in the preC/C region. First, the positive relationship between the preC/C mutation frequency and old age [wild type (36.9) vs. mutation (51.9), p = 0.001] suggests that the accumulation of preC/C mutations during the natural course of CHB contributes to the persistent infection of HBV in areas where vertical infection is predominant (Table 1).

Second, preC/C mutations in our cohort were distributed in a non-random manner, as shown in other studies (Fig. 1, Table 2) [36]. These preC/C mutations were found more frequently in immuno-active regions than in immuno-inactive regions (2.2% vs. 1.7%, p = 0.016), suggesting that the host immune pressure against the T cell is the major driving force of preC/C mutations [37], [38], [39]. Notably, a significant higher level of mutation rates in M2RR (2.3% vs. 1.7%, p = 0.009), but not in M1RR, as compared to in immuno-inactive region was found (Table 2), suggesting that M2RR, the target of the CD4 T helper cell, is more prone to mutations induced by the host immune response than M1RR, the target of the CD8 cytotoxic T cell [40].

Third, although differences in clinical factors apart from ALT level between patients with two different HBeAg serostatus were not found (Table S3), a significant correlation between the HBeAg-negative serostatus and preC/C mutations was observed in this study [HBe- (2.5%) vs. HBe+ (1.5%), p<0.001] (Table 4). This correlation strongly supports the findings of previous studies, where a high rate of HBV preC/C mutations emerges during the immune clearance phase, which is characterized by positive sera for HBeAg and an elevated ALT level [19], [22]. Our findings, in which only the mutations in M2RR [HBe- (3.0%) vs. HBe+ (1.7%), p<0.001] but not in the immuno-inactive region or M1RR (Table 4) were significantly related to the HBeAg-negative serostatus suggest that the accumulation of M2RR mutations within HBcAg contributes to the transfer from an immune-tolerance phase to an immune-clearance phase by breaking immune tolerance through immune evasion or a defect in the HBcAg assembly via mechanisms such as mutations in the I97 codon.

Finally, our data showed significant relationships between M2RR preC/C mutations, though not the immuno-inactive region or M1RR mutations, and HCC patients [HCC (2.7%) vs. comparison group (1.9%), p = 0.024] (Table 3), suggesting that immune evasion against the CD4 T cell via HBcAg mutation contributes to hepatocarcinogenesis. Therefore, inhibition of the cytotoxic T lymphocyte (CTL) function by the down-regulation of the CD4 T cell, rather than direct evasion of the CTL function, may be the principal strategy for HBV immune evasion.

Of seven mutations affecting HBeAg serostatus observed in this study, it is noteworthy that five (C-D32N/H, C-E43K, C-P50A/H/Y, C-A131G/N/P and C-S181H/P) in the C region may have been first introduced in relation to the HBeAg serostatus. In particular, to the best of our knowledge, two types of mutations (C-D32N/H, and C-E43K) related to the HBeAg positive serostatus have not been introduced to date in HBV variants. Interestingly, both mutations were not located at the regions of T or B cell epitopes, suggesting that their mutations are induced by other mechanisms, rather than by immune evasion. Generally, mutations associated with the HBeAg-negative serostatus are known to have the potential to be related to disease severity [19], [22]. However, no variant of those five related to the HBeAg-negative serostatus was significantly related to HCC, although preC-W28* tended to be linked to HCC (p = 0.093). The relationships between the five novel types of C mutations found in this study and the HBeAg serostatus and underlying molecular mechanisms require elucidation in the future.

Currently, relationships between the frequencies of the C region and the progression of liver disease have been assessed [24], [26]. However, a confirmative determination of a single amino acid change positively related to HCC has not been reported, although some C variants negatively related to HCC were recently reported [36] (Fig. 1). Of the five mutations in the C region positively related to HCC found in this study, the following three types, C-P5H/L/T, C-E83D, and C-I97F/L were introduced by other studies [41]. However, relationships between HCC and those three types have not yet been determined. To the best of our knowledge, the remaining two types (C-L100I and C-Q182K/*) may have been introduced for the first time in this study. It is noteworthy that four of five HCC-related mutations (80%) were located at M2RR. This strongly supports the above hypothesis that evasion against the CD4 T cell-mediated immune response, mainly via mutations in the “hot spot” region of aa residue 81–105, plays a role in the hepatocarcinogenesis of chronic patients infected with genotype C.

C-I97F/L, most frequently found in HCC-related mutations, is well known as the most frequently encountered HBcAg mutation, as mentioned in several studies [42], [43]. It has also been shown to lead to what is considered to be an immature secretion phenotype characteristic of the secretion of enveloped virions containing immature genomes. However, at present, it remains unclear if this variant contributes to the disease's severity, including HCC. The above issue should be addressed in future studies.

All of our sequence data were based on the direct sequencing protocol, which has the potential to underestimate deletions or point mutations at a level of less than 50% in each patient's viral quasispecies. Therefore, to resolve this concern, molecular based approaches such as real-time PCR (RT-PCR) based on fluorescence-resonance energy transfer (FRET) technology [44], [45], which can trace the HBV quasispecies, should be applied to the preC/C mutations, particularly those related to HCC or the HBeAg serostatus introduced in this study in future work.

Of note, high frequency of the lower level of HBV DNA in mutated strains than in wild strains were found in our cohort (Table 1, S2). It may be due to the inhibition of HBV replication by preC/C mutations. The identification of mutation types affecting HBV replication should also be done in the future study.

In conclusion, our data indicated that HBV variants in the C region, particularly in M2RR, may contribute to HCC progress in chronic patients infected with the genotype C via immune evasion against the CD4 T cell-mediated immune response. Furthermore, the identified HBcAg mutations related to HCC and affecting HBeAg serostatus have the potential to serve as diagnostic markers to detect early on the progression of liver disease, including HCC.

## Supporting Information

Figure S1 A phylogenic tree based on the sequence of the preC/C region from 70 and nine reference HBV strains. Genetic distances were estimated using the Kimura two-parameter matrix and the phylogenetic tree was constructed using the neighbor-joining method. The percentages indicated at the nodes represent bootstrap levels supported by 1000 re-sampled data sets. Bootstrap values of less than 50% are not shown. The bar indicates 1% estimated sequence divergence.
(TIF)

Table S1 Comparison of clinical features according to liver diseases.
(DOC)

Table S2 Comparison of the Clinical Features between patients with wild type and I97F/L.
(DOC)

Table S3 Comparison of Clinical Features of Patients according to HBeAg Serostatus.
(DOC)
