# Supplementary figures and images for: Naturally Occurring Precore/Core Region Mutations of Hepatitis B Virus Genotype C Related to Hepatocellular Carcinoma
Source: PLoS One. 2012 Oct 10;7(10):e47372. doi: 10.1371/journal.pone.0047372 (PMC3468518; doi:10.1371/journal.pone.0047372)

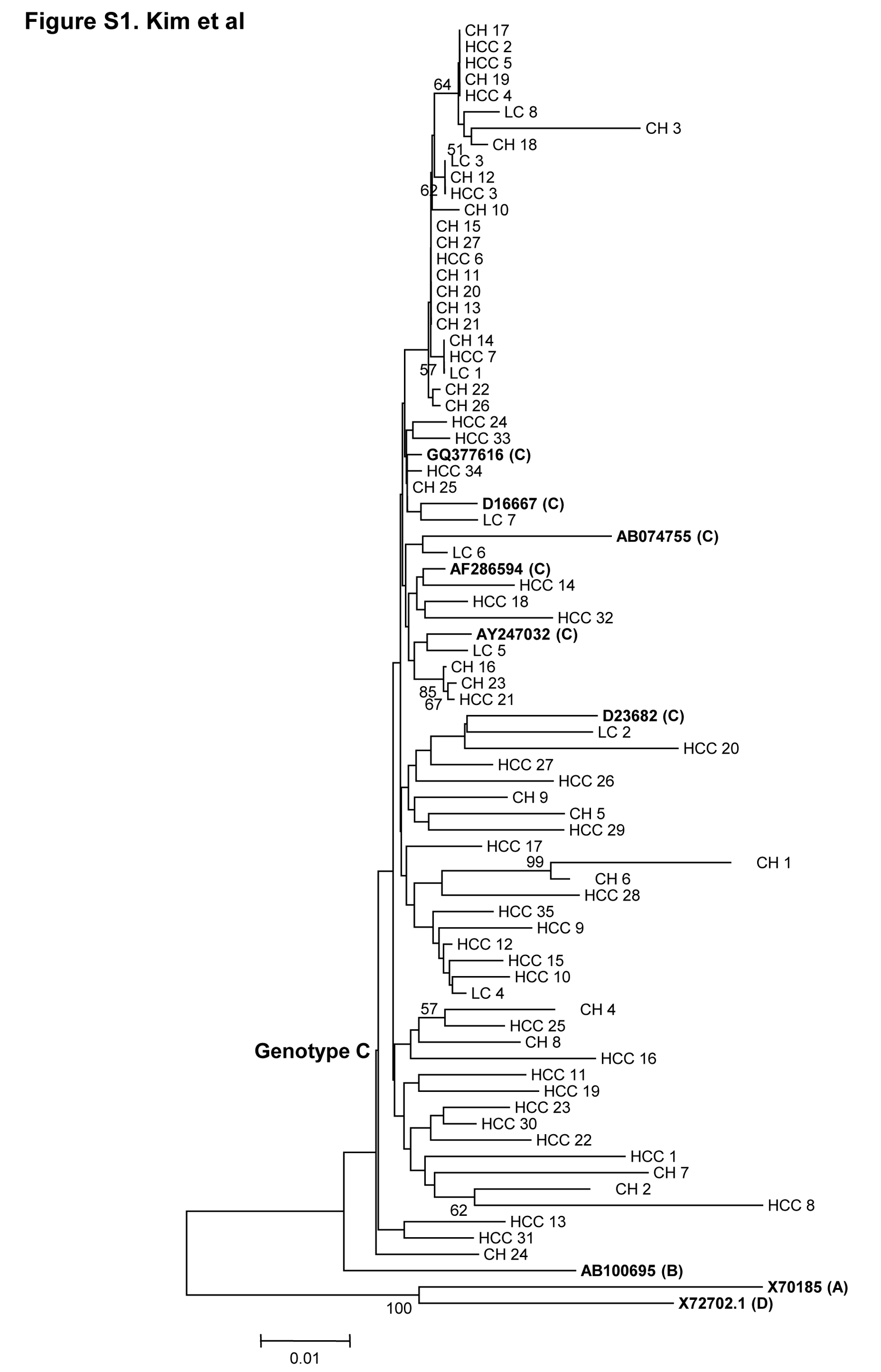

Supplement: Figure S1 — A phylogenic tree based on the sequence of the preC/C region from 70 and nine reference HBV strains. Genetic distances were estimated using the Kimura two-parameter matrix and the phylogenetic tree was constructed using the neighbor-joining method. The percentages indicated at the nodes represent bootstrap levels supported by 1000 re-sampled data sets. Bootstrap values of less than 50% are not shown. The bar indicates 1% estimated sequence divergence. (TIF) [file pone.0047372.s001.tif]
