# Supplementary material for: Naturally Occurring Precore/Core Region Mutations of Hepatitis B Virus Genotype C Related to Hepatocellular Carcinoma
Source: PLoS One. 2012 Oct 10;7(10):e47372. doi: 10.1371/journal.pone.0047372 (PMC3468518; doi:10.1371/journal.pone.0047372)
Supplement: Table S1 — Comparison of clinical features according to liver diseases. (DOC) [file pone.0047372.s002.doc]

Table S1. Comparison of clinical features according to liver diseases.

| Clinical factors | CH (n = 27) | LC (n = 8) | HCC (n = 35) |
| --- | --- | --- | --- |
| Age in years, mean ± SD | 43.3 + 15.9^a^ | 52.9 ± 16.8 | 54.1 ± 9.9 |
| Male (%) | 16 (59.3)^b^ | 5 (62.5)^c^ | 32 (91.4) |
| HBeAg-positive (%) | 11 (40.7) | 4 (50) | 20 (57.1) |
| ALT (IU/L), mean ± SD | 53.2 ± 63.6 | 198.0 ± 347.4 | 53.9 ± 38.7 |
| HBV-DNA (pg/ml) median (range) | 6.57E+06 (0-8.24E+07)^d^ | 6.05E+02 (0-3.03E+03) | 1.30E+00 (0-4.40E+01) |

CH: Chronic hepatitis, LC: Liver cirrhosis, HCC: Hepatocellular carcinoma

^a^ *p* = 0.003, compared with HCC; ^b^ *p* = 0.005, compared with HCC; ^c^ *p* = 0.033, compared with HCC , ^d^ *p* = 0.082, compared with HCC.
