# Supplementary material for: Naturally Occurring Precore/Core Region Mutations of Hepatitis B Virus Genotype C Related to Hepatocellular Carcinoma
Source: PLoS One. 2012 Oct 10;7(10):e47372. doi: 10.1371/journal.pone.0047372 (PMC3468518; doi:10.1371/journal.pone.0047372)
Supplement: Table S2 — Comparison of the Clinical Features between patients with wild type and I97F/L. (DOC) [file pone.0047372.s003.doc]

Table S2. Comparison of the Clinical Features between patients with wild type and I97F/L.

| Clinical factors | Wildtype (n = 53) | I97F/L (n = 17) | *P*-value |
| --- | --- | --- | --- |
| Age in years, mean ± SD | 47.3 ± 13.7 | 57.4 ± 13.0 | 0.01 |
| Male (%) | 40 (75.5) | 13 (76.5) | N.S^a^ |
| HBeAg-positive (%) | 30 (56.6) | 5 (29.4) | 0.093 |
| HCC (%) | 22 (41.5) | 13 (76.5) | 0.024 |
| ALT status^b^ (%) | 42 (79.2) | 11 (73.3) | N.S |
| HBV-DNA (pg/ml) median (range) | 3.34E+06 (0-8.24E+07) | 1.03E+04 (0-1.11E+05) | 0.083 |

^a^ N.S = Not significant

^b^ The number of patients whose ALT levels were greater than the upper limits of normal ALT levels for men (30 IU/L) and women (19 IU/L) [46].
