# Supplementary material for: Naturally Occurring Precore/Core Region Mutations of Hepatitis B Virus Genotype C Related to Hepatocellular Carcinoma
Source: PLoS One. 2012 Oct 10;7(10):e47372. doi: 10.1371/journal.pone.0047372 (PMC3468518; doi:10.1371/journal.pone.0047372)
Supplement: Table S3 — Comparison of Clinical Features of Patients according to HBeAg Serostatus. (DOC) [file pone.0047372.s004.doc]

Table S3. Comparison of Clinical Features of Patients according to HBeAg Serostatus.

| Clinical factors | HBe- (n = 35) | HBe+ (n = 35) | *P*-value |
| --- | --- | --- | --- |
| Age in years, mean ± SD | 50.0 ± 13.7 | 49.5 ± 14.8 | N.S^a^ |
| Male (%) | 27 (77.1) | 26 (74.3) | N.S |
| Liver disease (no.) CH:LC:HCC^b^ | 16:4:15 | 11:4:20 |  |
| ALT status^c^ (%) | 22 (66.7) | 31 (88.6) | 0.041 |
| HBV-DNA (pg/ml) median (range) | 5.91E+05 (0-2.03E+07) | 4.47E+06 (0-8.24E+07) | N.S |

^a^ N.S : Not significant

^b^ C: Carrier, CH: Chronic hepatitis, LC: Liver cirrhosis, HCC: Hepatocellular carcinoma

^c^ The number of patients whose ALT level were greater than upper limits of normal ALT for men (30 IU/L) and women (19 IU/L) [46].
